# Assessing Vehicle Profiling Accuracy of Handheld LiDAR Compared to Terrestrial Laser Scanning for Crash Scene Reconstruction

**DOI:** 10.3390/s21238076

**Published:** 2021-12-02

**Authors:** Jairaj Desai, Jidong Liu, Robert Hainje, Robert Oleksy, Ayman Habib, Darcy Bullock

**Affiliations:** 1 Joint Transportation Research Program, College of Engineering, Lyles School of Civil Engineering, Purdue University, West Lafayette, IN 47907, USA; liu2845@purdue.edu (J.L.); ahabib@purdue.edu (A.H.); darcy@purdue.edu (D.B.); 2 Tippecanoe County Sheriff’s Office, 2640 Duncan Road, Lafayette, IN 47904, USA; rwhainje@tippecanoe.in.gov; 3 Madison County Sheriff’s Department, 720 Central Ave., Anderson, IN 46016, USA; roleksy@madisoncounty.in.gov

**Keywords:** LiDAR, crash scene reconstruction, damage profiling, 3D scanning

## Abstract

Forensic crash investigation often requires developing detailed profiles showing the location and extent of vehicle damage to identify impact areas, impact direction, deformation, and estimated vehicle speeds at impact. Traditional damage profiling techniques require extended and comprehensive setups for mapping and measurement that are quite labor- and time-intensive. Due to the time involved, this damage profiling is usually done in a remote holding area after the crash scene is cleared. Light detection and ranging (LiDAR) scanning technology in consumer handheld electronic devices, such as smartphones and tablets, holds significant potential for conducting this damage profile mapping in just a few minutes, allowing the mapping to be conducted at the scene before the vehicle(s) are moved. However, there is limited research and even scarcer published literature on field procedures and/or accuracy for these emerging smartphones and tablets with LiDAR. This paper proposes a methodology and subsequent measurement accuracy comparisons for survey-grade terrestrial laser scanning (TLS) and handheld alternatives. The maximum root mean square error (RMSE) obtained for profile distance between handheld (iPad) and survey-grade TLS LiDAR scans for a damaged vehicle was observed to be 3 cm, a level of accuracy that is likely sufficient and acceptable for most forensic studies.

## 1. Background

For severe crashes, agencies investigating the crash often take vehicle position measurements at the scene prior to vehicle recovery and scene clearance. The Federal Highway Administration’s (FHWA) Traffic Incident Management (TIM) program observed that primary crashes increase the likelihood of a secondary crash occurring and may impact the safety of public safety agencies and first responders trying to document and clear the scene to restart traffic flow [1]. The FHWA also identified that the possibility of a secondary crash rises by 2.8% for every minute a primary crash incident continues to impact the public right of way [2]. As a result, there is strong national interest in identifying new opportunities to help first responders safely manage and clear crashes as quickly as practical. Several recent efforts have examined the use of unmanned aircraft systems (UAS) to map and document crash scenes to accelerate scene clearance and reduce risk to first responders on scene [3,4]. However, documenting crush damage on individual vehicles involved in an incident requires close quarter scans where UAS maneuverability may prove challenging.

There are trade-offs between how long to devote to on-scene measurements vs. quick scene clearance and subsequent measurements of the vehicle at a holding facility at a later date. In general, the more measurements that are performed on-site, the more confident one is that additional damage did not occur during recovery towing. Identifying emerging technologies to provide fast and accurate characterization of the vehicle damage on-site reduces this ambiguity during subsequent forensic crash investigation work.

## 2. Measurements Characterizing Vehicle Deformation

**Figure 1:**
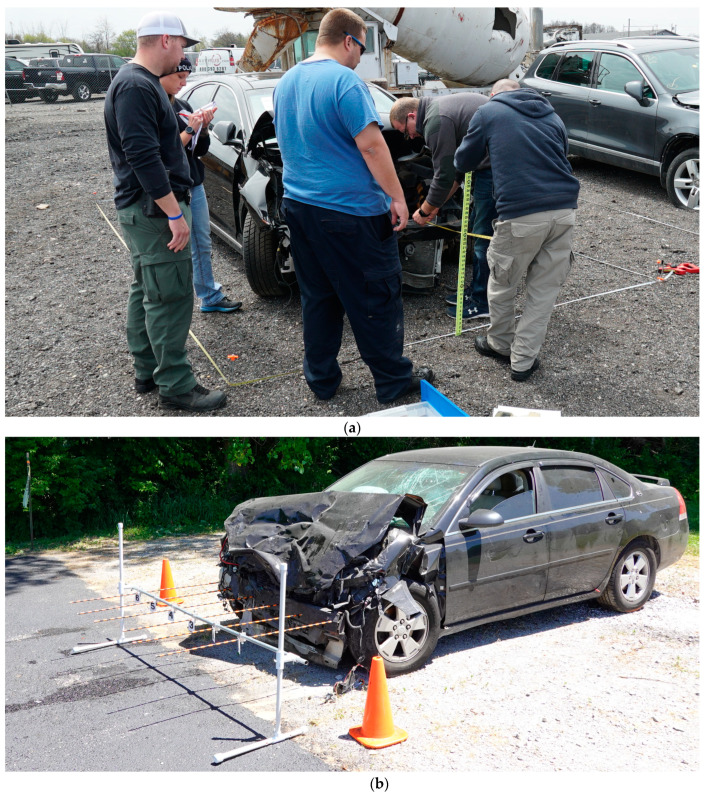
Traditional vehicle profiling techniques: (**a**) Tape measure; (**b**) PVC jig.

Measurement protocols for defining damage profiles in crash vehicles and their use for estimating vehicle speeds at impact have been well-documented in existing literature for traditional measurement techniques involving the use of a set of tape measures (Figure 1a), plumb bobs, jigs, (Figure 1b), datum strings, plywood board method or the plastic sheet method [5,6]. However, these profiling techniques may involve human errors due to transcription and require significant time to obtain comprehensive data for a 3D model. 

Studies in the past have explored the accuracy of photogrammetric measurements compared to traditional hands-on methods such as tape measures, plumb bobs, and crush PVC jigs (such as those depicted in Figure 1a,b) and found photogrammetry to have slightly higher accuracy although requiring shorter scan times [7]. However, photogrammetric reconstruction might fail in identifying conjugate features in overlapping images due to insufficient overlap and/or excessive variation in camera-to-object distance. While photogrammetry has the potential to provide detailed visual information on damaged vehicles which may provide contextual evidence for a crash scene, LiDAR is perhaps even more accurate and is capable of directly providing depth information in a mapping environment. Researchers have shown how terrestrial laser scanning methods employing LiDAR can be applied towards vehicle crush measurements [8]. Techniques involving the use of total stations [9] or terrestrial LiDAR scanners require extended setups and multiple scans to accurately capture crush damage profiles. However, equipment such as total stations or laser/LiDAR scanners are cost prohibitive for public safety agencies to deploy with every officer and they present operational challenges in portability in situations requiring quick deployment due to logistics associated with bringing them to the crash scene and having trained officers available to operate the equipment [10,11]. Research has also shown that iOS-based LiDAR 3D scan measurements are repeatable with low standard deviations, thus lending further confidence to the possible widespread adoption of these techniques across the emergency response domain [12].

## 3. Motivation

The motivation for this research was to develop an evaluation protocol and apply that protocol to determine if handheld tablets or smartphones could be used to perform a 3D scan of a vehicle that would provide similar accuracy as survey-grade LiDAR equipment in less time. If similar accuracies can be obtained and scan times are significantly shorter, it would be desirable to do these scans on-scene before vehicles are moved, to capture evidence as representative of the original incident as possible. Even if survey-grade TLS scans are performed at a later date, having the on-scene 3D scans that align closely with TLS scans reduces any ambiguity or doubt that some damage may have occurred after the vehicle was moved from the scene.

## 4. Equipment and Data Collection Procedure

**Figure 2:**
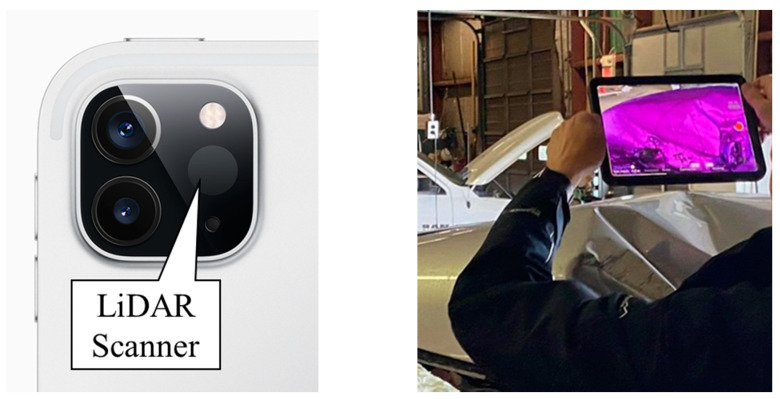
Handheld LiDAR scanning equipment.

**Figure 3:**
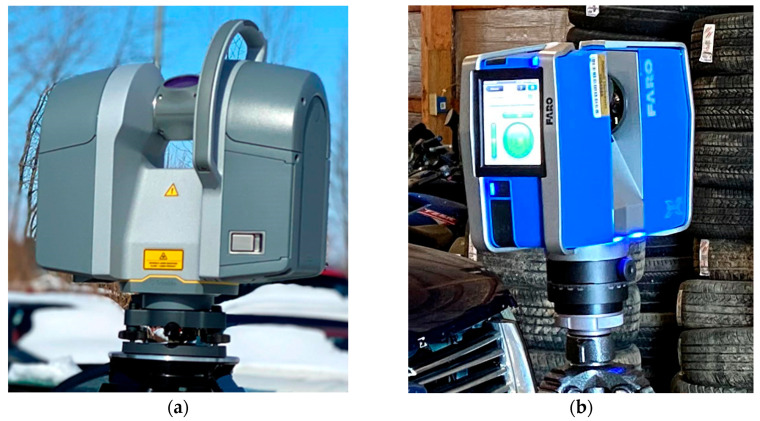
Terrestrial Laser Scanning (TLS) equipment: (**a**) Trimble TX8b Laser Scanner; (**b**) FARO Focus 3D X330 Laser Scanner.

The data acquisition equipment used in this study were an iPad Pro (2nd generation) [13] (Figure 2), and two independent terrestrial laser scanners: FARO Focus 3D X330 (Figure 3a) and Trimble TX8b (Figure 3b). The iPad handheld LiDAR scanner works at a range of up to 5 m. The FARO Focus 3D X330 laser scanner can scan up to 976,000 points per second with a maximum range of 330 m and a range accuracy of ±2 mm [14]. The Trimble TX8b laser scanner can scan up to one million points per second with a maximum range of 120 m and a range accuracy better than ±2 mm [15].

For the handheld LiDAR data collection, multiple passes were conducted by walking around the damaged vehicle at varying distances and elevations from the surface with the handheld device’s LiDAR scanner directly pointing at the vehicle. The scan required a total time of approximately 15 min to ensure sufficient coverage of all surfaces of the vehicle captured for this study.

**Figure 4:**
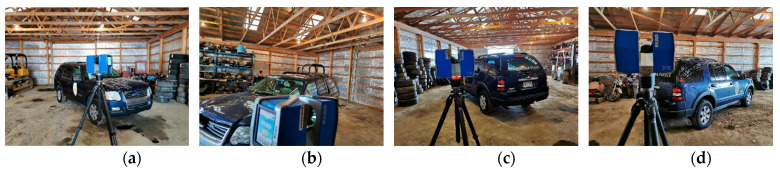
TLS equipment setup: (**a**) Setup 1; (**b**) Setup 2; (**c**) Setup 3; (**d**) Setup 4.

**Figure 5:**
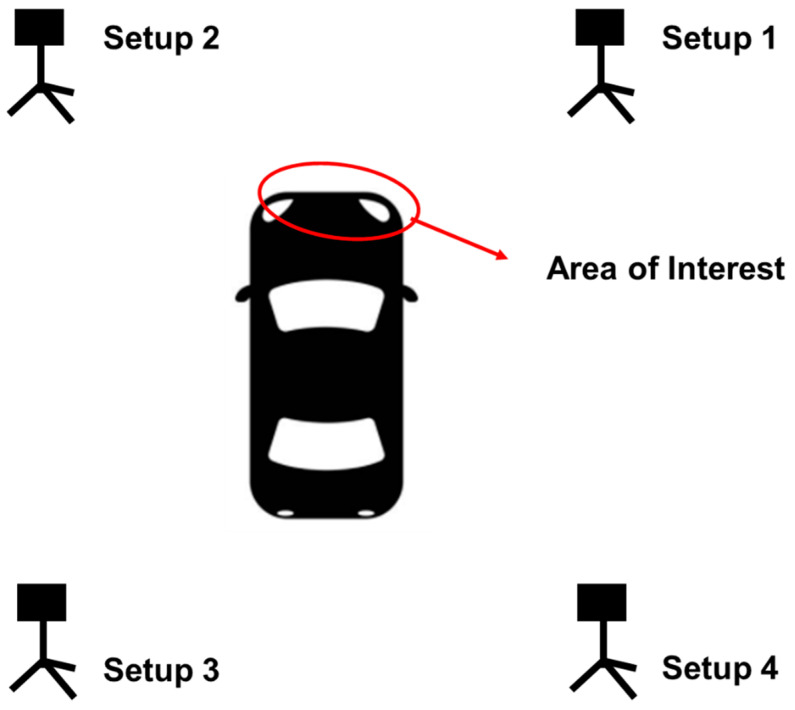
TLS equipment setup locations around damaged vehicle.

For the TLS survey, each scanner required four scans in order to provide a complete coverage of the vehicle. Figure 4 shows the FARO stations set up. Each TLS scan took about twenty minutes for a total of three hours considering the time for scanners’ move and setup between the different locations. A representative diagram of the four locations where TLS stations were set up around the damaged vehicle is depicted in Figure 5.

## 5. Methodology

**Figure 6:**
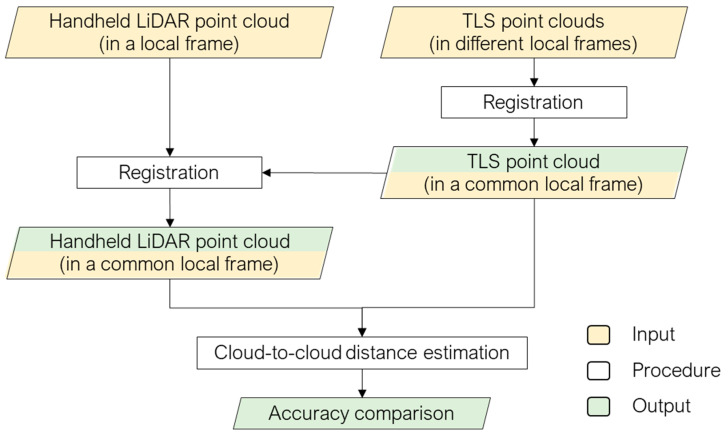
Workflow of the proposed point cloud registration and comparison strategy.

In this study, the quality of handheld LiDAR data for damage profile mapping is evaluated based on a comparison between the handheld LiDAR generated point cloud and the point cloud generated by the TLS. A systematic cloud-to-cloud comparison is essential when evaluating any new sensing technology in order to instill user confidence in measurement accuracy for widespread adoption. The methodologies proposed herein define the fundamental techniques for evaluating the sensor accuracy. Figure 6 illustrates the workflow, which includes two major components: point cloud registration and cloud-to-cloud distance estimation. 

In this study, data acquisition and post-processing of handheld LiDAR is based on the 3D Scanner AppTM [16] application available for the LiDAR capable iPad, and the output is a color-coded point cloud by spectral information (RGB) in a unified reference frame established by the scanning application. For TLS, the point cloud acquired by each scan is available in a different local reference frame that is defined by the scan location/setup. A registration is required to bring the point clouds from the different scans to a common local reference frame. In order to enable a direct comparison, the survey-grade and handheld LiDAR point clouds should be registered to a common reference frame. The following subsections describe the point cloud registration and comparison strategies.

### 5.1. Point Cloud Registration

A coarse registration followed by a fine registration is performed to align the point clouds from different scans/systems. The first registration pass provides an initial estimate of the transformation parameters for rough alignment of the point clouds. The second registration pass fine-tunes the transformation parameters to ensure better, more precise alignment among the point clouds. 

In this study, coarse registration is conducted by manually identifying conjugate point pairs in areas of overlap among the point clouds. For fine registration, the iterative closest projected point (ICPP) approach [17] is adopted. The ICPP approach starts with establishing point correspondences between two point clouds—one is selected as a reference and another as a source. For a point *a* in the source cloud, the ICPP finds its three closest points, *p*, *q*, and *i*, in the reference cloud, and forms a triangular patch. The source point *a* is then projected onto the triangular patch. If the projected point *b* falls within the patch, it will be considered as a corresponding point to the source point *a* only if their separation is below a user-defined threshold. Once the point-to-point correspondence is established, the algorithm estimates the parameters of a rigid body transformation between the two point clouds. The estimated transformation parameters are then applied to the source cloud to refine the alignment between the source and reference clouds. This process (i.e., establishing point pairs as well as estimating and applying the transformation parameters) is repeated until preset convergence accuracy requirements are met. 

**Figure 7:**
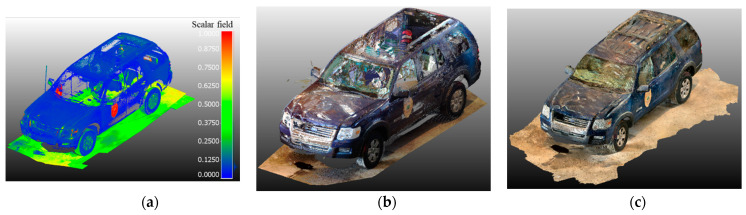
TLS and Handheld Scan Results: (**a**) Trimble Scan colored by intensity; (**b**) FARO Scan colored by RGB; (**c**) iPad Scan point cloud colored by RGB.

The above-mentioned point cloud registration strategy is performed for aligning: (i) point clouds from different TLS scans and (ii) handheld LiDAR and TLS point clouds, as depicted in Figure 6. The point clouds after registration from the Trimble, FARO, and handheld LiDAR are shown in Figure 7.

### 5.2. Cloud-to-Cloud Distance Estimation

**Figure 8:**
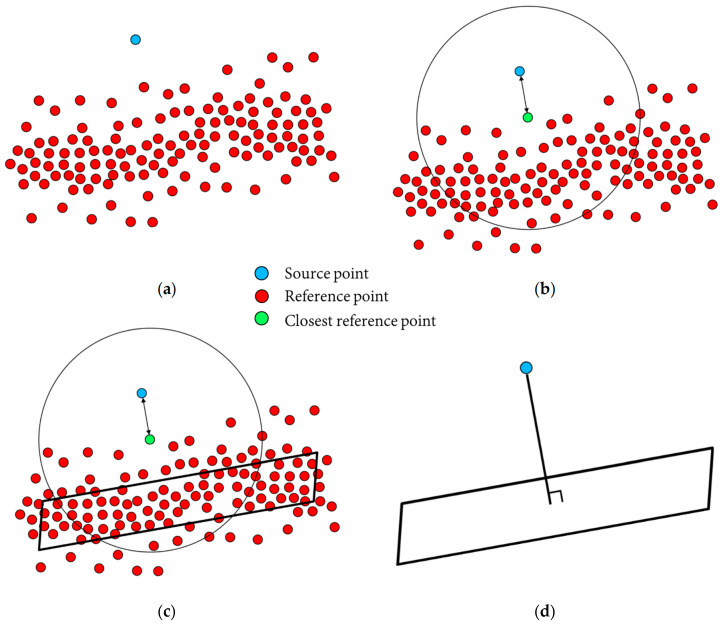
Schematic illustration of computing distance between two point clouds: (**a**) source point (blue) and reference points (red), (**b**) closest reference point (green) and corresponding spherical region for extracting neighboring point, (**c**) iterative fitted plane, and (**d**) distance between source point and corresponding plane in the reference point cloud.

Once the point clouds from handheld LiDAR and TLS (FARO, Trimble) are registered, the discrepancy between the two point clouds is estimated by calculating the cloud-to-cloud distance using the strategy proposed in [18], as illustrated in Figure 8. For a given point in the source point cloud (blue point in Figure 8a), its closest point in the reference point cloud is first identified, as shown by the green point in Figure 8b. Then, a spherical region with a pre-defined radius centered at the closest point is created (Figure 8b). An iterative plane fitting is conducted using the points in the reference point cloud within the spherical region, as shown in Figure 8c. The objective of the iterative plane fitting is removing outlier points that might not belong to the planar neighborhood in Figure 8c. Lastly, the normal distance between the selected source point and corresponding plane is computed (Figure 8d). The normal distance can be used as a coloring scalar value to qualitatively illustrate the cloud-to-cloud separation. Moreover, the mean, standard deviation, and root mean square error (RMSE) of the estimated normal distances are used to quantitatively describe the closeness of the source and reference point clouds after the fine registration process. The results that follow show cloud-to-cloud distance estimation between the FARO and handheld LiDAR scans as well as the Trimble and handheld LiDAR scans separately.

## 6. Comparison of Handheld LiDAR and TLS Models

Qualitative and quantitative comparisons between handheld LiDAR and TLS data were carried out to assess the quality of point clouds acquired by the two acquisition systems. The handheld LiDAR and TLS point clouds were selected as the source and reference, respectively. The cloud-to-cloud distance between points in the handheld LiDAR point cloud and each TLS dataset was calculated, henceforth referred to as residuals.

**Figure 9:**
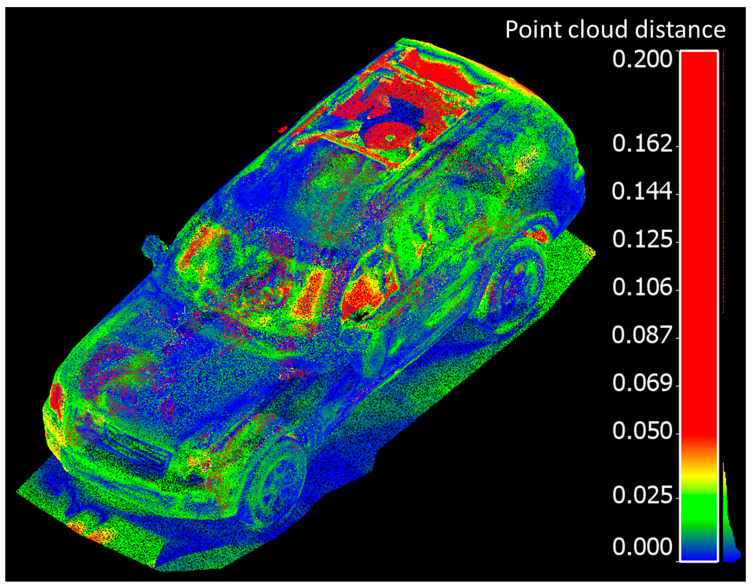
Point cloud distance comparisons between handheld LiDAR and FARO scans (meters).

**Figure 10:**
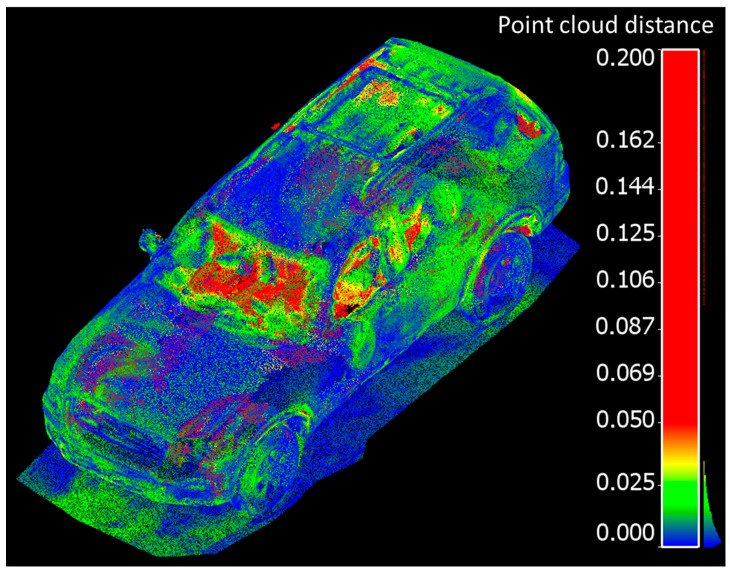
Point cloud distance comparisons between handheld LiDAR and Trimble scans (meters).

- The residuals between the FARO and handheld LiDAR scans are shown in Figure 9.
- The residuals between the Trimble and handheld LiDAR scans are shown in Figure 10.

Overall, point clouds from both techniques and for both sets of comparisons are in good agreement over solid surfaces of the vehicle. The larger discrepancies (shown in red), were typically associated with some units “seeing through glass” and others, not. The mean, standard deviation, and root mean square error (RMSE) of the cloud-to-cloud distance between the handheld LiDAR and FARO point clouds is 1.7 cm, 1.6 cm, and 2.3 cm, respectively. Similarly, the mean, standard deviation, and root mean square error (RMSE) of the cloud-to-cloud distance between the handheld LiDAR and Trimble point clouds is 1.6 cm, 1.5 cm, and 2.2 cm, respectively.

To further examine the details captured by the LiDAR scan, ten transverse cross-sectional and four longitudinal cross-sectional profiles were extracted from the handheld LiDAR point cloud.

**Figure 11:**
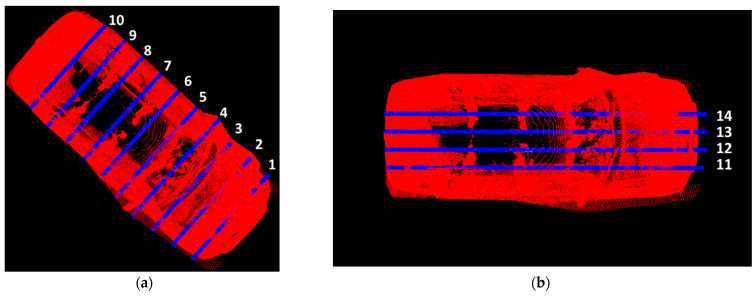
Transverse and Longitudinal Cross-sectional vehicle profiles to calculate cloud-to-cloud distance between FARO (red) and handheld LiDAR profiles (blue): (**a**) Transverse Cross-sectional profiles; (**b**) Longitudinal Cross-sectional profiles.

**Figure 12:**
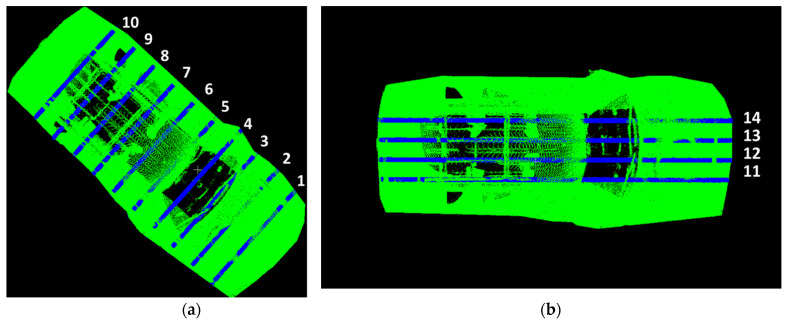
Transverse and Longitudinal Cross-sectional vehicle profiles to calculate cloud-to-cloud distance between Trimble (green) and handheld LiDAR profiles (blue): (**a**) Transverse Cross-sectional profiles; (**b**) Longitudinal Cross-sectional profiles.

- The transverse and longitudinal cross sections for the FARO scanner are shown in Figure 11a,b, respectively.
- The transverse and longitudinal cross sections for the Trimble scanner are shown in Figure 12a,b, respectively.

The cross sections in Figure 11 and Figure 12 were used to compute cloud-to-cloud distance estimates obtained from the handheld LiDAR scan.

**Figure 13:**
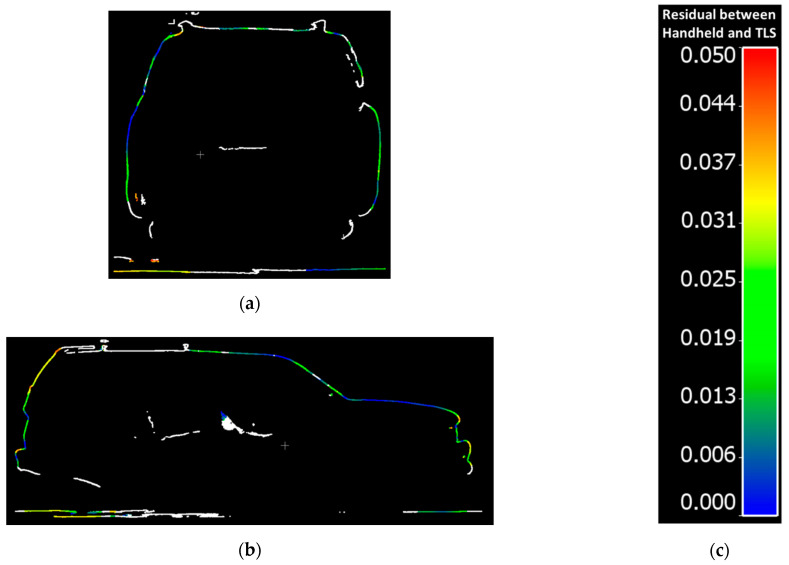
Cloud-to-cloud distance residual samples for Transverse and Longitudinal Cross-sectional vehicle profiles for FARO and handheld LiDAR: (**a**) Transverse Cross-sectional profile 7; (**b**) Longitudinal Cross-sectional profile 13; (**c**) Color scale for Residual between Handheld and TLS.

**Figure 14:**
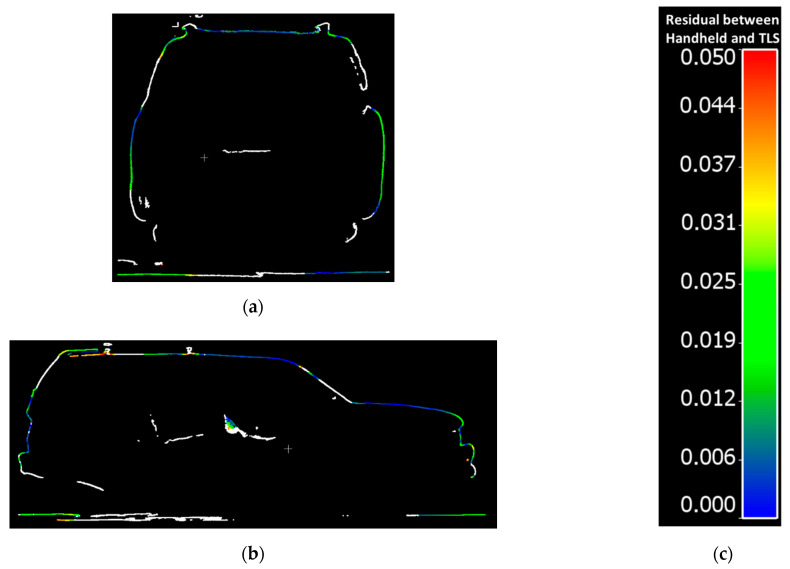
Cloud-to-cloud distance residual samples for Transverse and Longitudinal Cross-sectional vehicle profiles for Trimble and handheld LiDAR: (**a**) Transverse Cross-sectional profile 7; (**b**) Longitudinal Cross-sectional profile 13; (**c**) Color scale for Residual between Handheld and TLS.

- Figure 13a,b show side views of sample transverse cross-sectional and longitudinal cross-sectional profiles from the handheld LiDAR data colored by cloud-to-cloud distance with the FARO scans, a scale for which is included in Figure 13c.
- Figure 14a,b show side views of sample transverse cross-sectional and longitudinal cross-sectional profiles from the handheld LiDAR data colored by cloud-to-cloud distance with the Trimble scans, a scale for which is included in Figure 14c.

Points with cloud-to-cloud distances larger than 5 cm are colored in white. As can be seen in Figure 13 and Figure 14, the areas with larger discrepancies are inside the vehicle. Those discrepancies occur because the TLS (FARO, Trimble), with limited scan locations, could only cover the exterior of the vehicle. The handheld scanner, on the contrary, can move around the vehicle and had better visibility of the interior of the vehicle. 

Table 1 reports the statistics of the cloud-to-cloud distance between the FARO scans and handheld LiDAR for each of the 14 profiles, including the mean, standard deviation, and RMSE. Table 2 reports those same statistics for the Trimble scans. According to the RMSE in both tables, the point clouds from handheld LiDAR and both TLS equipment options (FARO, Trimble), are in good agreement within a range of 3 cm.

## 7. Conclusions and Future Scope

This paper proposes a methodology and subsequent measurement accuracy comparisons for survey-grade terrestrial laser scanning (TLS) and handheld alternatives. An iPad Pro (handheld) and two different survey-grade TLS LiDAR units were used to profile a damaged vehicle for this study. Point cloud comparisons between the handheld and terrestrial LiDAR scanning techniques for a damaged vehicle show good agreement. The methodology presented in this study for point cloud comparison will provide a strong foundation for future evaluations of remote sensing alternatives by public safety professionals as more and more consumer grade LiDAR technologies become increasingly available in off-the-shelf electronic handheld devices. The maximum root mean square error (RMSE) obtained for profile distance between handheld and TLS scans for a damaged vehicle was observed to be 3 cm, a level of accuracy that is likely sufficient and acceptable for most forensic studies. This level of accuracy is sufficient for crash scene reconstruction as it is similar to the measurement error of currently used technology. Handheld scanning methods provide significant time savings compared to TLS and traditional vehicle profiling methods (minutes compared to hours). Secondly, handheld scanners afford first responders and reconstructionists alike the ability to reach and scan traditionally unreachable locations of damaged vehicles such as the interiors or the undercarriage, thus ensuring a comprehensive scan of all observed damage, and not being limited by the range or maneuverability of traditional terrestrial laser scanners. Adoption of these techniques by public safety agencies and first responders has the potential to aid in faster scene clearance and reduce secondary crashes. Furthermore, on-scene documentation of vehicle damage profile reduces the ambiguity as to whether the damage occurred during the crash, or after the vehicle was moved to an off-site yard.

Future research in this domain will involve evaluating the performance of point clouds derived from RGB images captured by smartphone devices using Structure from Motion (SfM) strategies. Additionally, SfM point clouds derived from unmanned aerial vehicle (UAV) imagery documenting crash scenes may further be augmented by handheld LiDAR data.

## Figures and Tables

**Table 1:** Statistics of cloud-to-cloud distance between the handheld LiDAR profiles and FARO data.

Profile Type	Profile ID	Mean (cm)	Std. Dev. (cm)	RMSE (cm)
Transverse Cross section (Figure 11a)	1	1.9	2.4	3.0
	2	1.1	1.4	1.8
	3	1.5	1.9	2.4
	4	1.2	1.3	1.7
	5	1.1	0.9	1.4
	6	1.5	1.6	2.2
	7	1.9	1.7	2.6
	8	2.0	2.0	2.9
	9	1.7	1.6	2.4
	10	2.1	1.6	2.7
Longitudinal Cross section (Figure 11b)	11	2.2	1.5	2.6
	12	2.0	1.6	2.5
	13	1.7	1.3	2.2
	14	1.9	1.6	2.5

**Table 2:** Statistics of cloud-to-cloud distance between the handheld LiDAR profiles and Trimble data.

Profile Type	Profile ID	Mean (cm)	Std. Dev. (cm)	RMSE (cm)
Transverse Cross section (Figure 12a)	1	1.6	2.0	2.6
	2	0.9	1.3	1.6
	3	1.7	1.8	2.4
	4	1.1	0.8	1.4
	5	0.9	0.8	1.3
	6	1.3	1.2	1.8
	7	1.6	1.6	2.3
	8	2.0	1.7	2.6
	9	1.9	1.7	2.6
	10	2.2	1.5	2.7
Longitudinal Cross section (Figure 12b)	11	2.0	2.1	2.4
	12	1.9	1.6	2.5
	13	1.5	1.5	2.1
	14	1.2	1.2	1.7

## Data Availability

Not applicable.
